# Association of changes of pulse wave velocity and augmentation index after isometric handgrip exercise with coronary lesion extent and revascularization

**DOI:** 10.1186/s40885-021-00163-5

**Published:** 2021-05-01

**Authors:** Seong Taeg Kim, Yeekyoung Ko, Jong-Wook Beom, Ki Yung Boo, Jae-Geun Lee, Joon-Hyouk Choi, Song-Yi Kim, Seung-Jae Joo

**Affiliations:** 1 Department of Internal Medicine, Jeju National University Hospital, 15 Aran 13-gil, Jeju City, Jeju Province 63241 Republic of Korea; 2 Department of Internal Medicine, Jeju National University School of Medicine, 15 Aran 13-gil, Jeju City, Jeju Province 63241 Republic of Korea; 3 Institute of Medical Science, Jeju National University, 15 Aran 13-gil, Jeju City, Jeju Province 63241 Republic of Korea

**Keywords:** Arterial stiffness, Pulse wave velocity, Pulse wave analysis, Isometric exercise, Percutaneous coronary intervention

## Abstract

**Background:**

Arterial stiffness is associated with myocardial ischemia and incident coronary artery disease (CAD), and indexes of arterial stiffness are usually increased in patients with CAD. However, these indexes are often increased in elderly without CAD. Arterial stiffness in patients with CAD may become more evident after isometric handgrip exercise which increases systolic pressure and ventricular afterload. We investigated the association of the change of stiffness indexes after isometric handgrip exercise with the lesion extent of CAD and the necessity for coronary revascularization.

**Methods:**

Patients who were scheduled a routine coronary angiography via a femoral artery were enrolled. Arterial waveforms were traced at aortic root and external iliac artery using coronary catheters at baseline and 3 min after handgrip exercise. Augmentation index (AIx) was measured on the recorded aortic pressure waveform, and pulse wave velocity (PWV) was calculated using the ECG-gated time difference of the upstroke of arterial waveforms and distance between aortic root and external iliac artery.

**Results:**

Total 37 patients were evaluated. Both PWV and AIx increased after handgrip exercise. ΔPWV was significantly correlated with ΔAIx (*r* = 0.344, *P* = 0.037). Patients were divided into higher and lower ΔPWV or ΔAIx groups based on the median values of 0.4 m/sec and 3.3%, respectively. Patients with higher PWV had more 2- or 3-vessel CAD (69% vs. 27%, *P* = 0.034), and underwent percutaneous coronary intervention (PCI) more frequently (84% vs. 50%, *P* = 0.038), but higher ΔAIx was not associated with either the lesion extent or PCI. Area under curve (AUC) of ΔPWV in association with PCI by C-statistics was 0.70 (95% confidence interval [CI] 0.51–0.88; *P* = 0.056). In multiple logistic regression analysis, ΔPWV was significantly associated with PCI (odds ratio 7.78; 95% CI 1.26–48.02; *P* = 0.027).

**Conclusions:**

Higher ΔPWV after isometric handgrip exercise was associated with the lesion extent of CAD and the necessity for coronary revascularization, but higher ΔAIx was not.

## Background

Normal or accelerated vascular aging and hypertension are two main factors determining arterial stiffness because they are basically related to the change of the arterial media [1]. However, diabetes, dyslipidemia, and smoking in addition to vascular aging and hypertension, all of which are shared common risk factors for coronary artery disease (CAD), may also change the mechanical properties of the arterial wall to make a stiff artery [2]. In a stiff arterial system, the velocities of the incident flow and the backward reflection flow are rapid enough for the early return of reflected waves during arterial systole rather than diastole, and the incident and reflected pressure waves are summed to increase aortic or central systolic pressure, but decrease central diastolic pressure, resulting in widened central pulse pressure [3]. Augmented central systolic pressure increases ventricular afterload and myocardial oxygen demand, and lowered diastolic pressure decreases coronary blood perfusion: the net results are myocardial ischemia and ventricular dysfunction [3–6]. For this reason, a stiff arterial system is associated with incident CAD, heart failure or stroke [7–9], and indexes of stiffness are related to the presence and severity of CAD [5]. Therefore, a stiff large artery is another feature of patients with CAD.

One of the popular methods evaluating arterial stiffness is measuring pulse wave velocity (PWV), a speed of an arterial pulsation through the arterial tree, usually between carotid and femoral arteries [6]. A number of studies reported that PWV was increased in patients with CAD, and correlated with the severity of coronary atherosclerosis [5, 10–14]. Increased aortic PWV (> 10 m/sec) is considered as a marker for detecting hypertension-mediated organ damage in guidelines for the management of arterial hypertension [15, 16]. Another method for measuring the arterial stiffness is central pulse wave analysis. Augmentation by the summation of incident and reflected waves in the aortic pressure waveform is expressed as augmentation pressure (AP) or augmentation index (AIx). AIx is a percentage of AP on aortic pulse pressure [3, 6]. The earlier studies showed that AIx was increased in patients with CAD and was associated with the severity of CAD, especially in younger patients (< 60 years of age) [10, 17, 18]. We already reported that AIx was negatively correlated with minimal luminal area of coronary atherosclerosis measured by intravascular ultrasound, and was associated with coronary revascularization [19]. However, other studies showed no association of AIx with CAD, especially in elderly patients [8, 12, 20].

Increased PWV or AIx in patients with CAD may become more evident with a maneuver that influences the velocities of incident and reflected pressure waves. Isometric handgrip exercise, which increases systolic blood pressure (BP) and ventricular afterload [21–23], is a suitable and easily applicable tool for this purpose. The changes of PWV and AIx after isometric handgrip exercise may unmask the lesion extent and the necessity for coronary revascularization in elderly patients with CAD. In this study, the association of the change of PWV or AIx after isometric handgrip exercise with the lesion extent of CAD and the necessity for coronary revascularization was investigated.

## Methods

### Study patients

Patients who were scheduled a routine coronary angiography (CAG) via a femoral artery for the evaluation of the coronary atherosclerosis, and agreed to participate in the study were enrolled. Patients with acute coronary syndrome, or valvular heart disease were excluded. CAD was defined as ≥70% stenosis of the luminal diameter by the visual estimation of the operator in at least one major epicardial coronary artery or a past medical history of coronary revascularization. The extent of coronary atherosclerosis was classified as 1-, 2- or 3-vessel disease by the number of the major epicardial coronary arteries with a significant stenosis. Patients with a significant stenosis in the left main coronary artery were counted as having 2-vessel disease.

The study protocol was approved by the institutional review board of Jeju National University Hospital. Written informed consents were obtained from participating patients or legal representative.

### Study protocol

The maximal voluntary forearm contraction power was measured with a JAMAR dynamometer (Sammons Preston Rolyan, Nottinghamshire, UK), and a submaximal target at 30 ~ 40% of maximal handgrip power was used for 3-min isometric handgrip exercise.

**Fig. 1:**
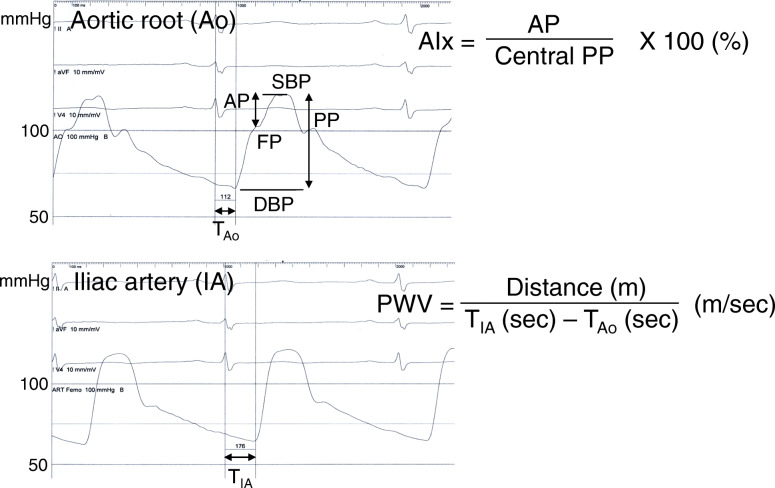
Measurements of augmentation index (AIx), and pulse wave velocity (PWV). Forward pressure (FP) was measured at a merging point of the forward and the reflected waves on the recorded aortic pressure waveform. AIx was defined as augmentation pressure (AP) divided by central pulse pressure (PP), and expressed as a percentage. PWV was calculated using the ECG-gated time difference of the upstroke of the arterial waveforms between the aortic root (T_AO_) and the external iliac artery (T_IA_), and the distance between them determined by a tape measure of the catheter length after removal of a coronary catheter. DBP; diastolic blood pressure, SBP; systolic blood pressure

After routine CAG, arterial pressure waveforms were traced using a right coronary catheter and a fluid-filled pressure transducer system. Central arterial waveforms were recorded at the aortic root and traced before and at 1, 2 and 3 min after handgrip exercise. Forward pressure was measured at a merging point of the forward and the reflected waves on the recorded aortic pressure waveform. Augmentation pressure was defined as maximal central systolic pressure minus forward pressure. AIx was defined as augmentation pressure divided by central pulse pressure and expressed as a percentage (Fig. 1). Peripheral arterial waveforms were recorded at the external iliac artery before and after 3-min handgrip exercise. The ECG-gated time difference (ΔTime) of the upstroke of the central and peripheral arterial waveforms was measured. The distance (D) between the aortic root and the external iliac artery was determined by a tape measure of the catheter length from the tip to the entry point at an arterial sheath minus the length of an arterial sheath (12 cm). PWV was defined as D divided by ΔTime (Fig. 1) [24].

### Statistics

Data were expressed as mean ± standard deviation for continuous variables, and as number (percentage) for categorical variables. Changes of hemodynamic parameters after handgrip exercise were analyzed using paired t-test, or where appropriate, Wilcoxon signed rank test. The association between changes of PWV (ΔPWV) and AIx (ΔAIx) after handgrip exercise was evaluated using correlation analysis. Patients were divided into higher and lower ΔPWV or ΔAIx groups based on the each median value. Data between groups were compared using unpaired t-test for continuous variables, and chi-square test for categorical variables. The association between higher ΔPWV or ΔAIx and coronary revascularization was evaluated using C-statistics and multiple logistic regression analysis after adjusting for age, gender, height, body mass index (BMI), hypertension, diabetes mellitus (DM) and chronic kidney disease (CKD). CKD was defined as estimated glomerular filtration rate < 60 mL/min/1.73m^2^ by Modification of Diet in Renal Disease equation.

All statistical analyses were performed with the statistical package SPSS version 23 (IBM Co, Armonk, NY, US). Clinical significance was defined as *P* < 0.05.

## Results

Total 42 patients were enrolled. After excluding 4 patients without a definite inflection point of augmentation pressure on the central arterial waveform and 1 patient without a peripheral arterial waveform, final 37 patients were evaluated. The mean age of study patients was 63.3 ± 9.4 years (range 41–82 years), 24 patients (65%) were older than 60 years, 26 patients (70%) had hypertension, and 27 patients (73%) had CAD. Percutaneous coronary intervention (PCI) was conducted in 25 patients (68%): 1 year ago in 20 patients and on the day of the study in 5 patients. One patient underwent both PCI and coronary artery bypass graft.

**Fig. 2:**
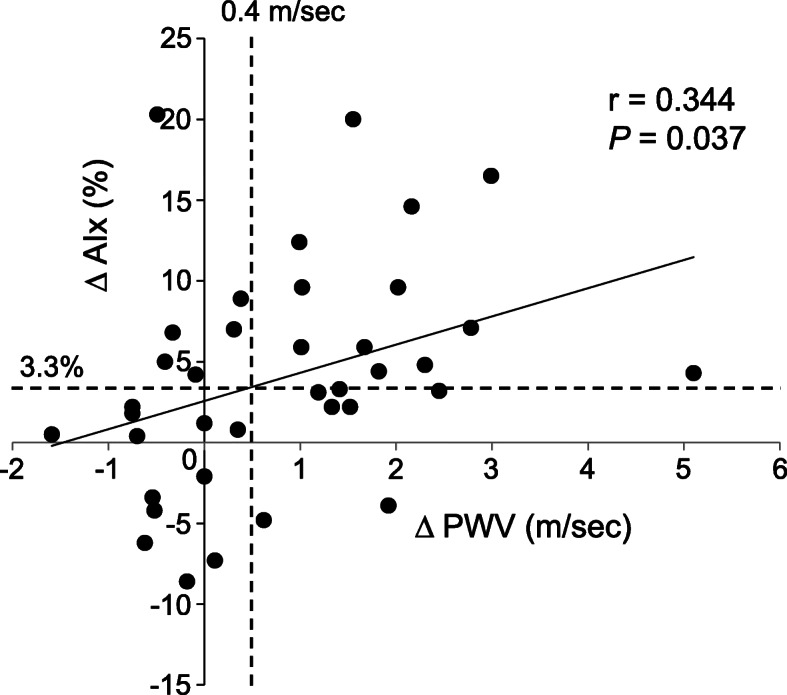
Correlation between changes of pulse wave velocity (ΔPWV) and augmentation index (ΔAIx) after isometric handgrip exercise. The median values of ΔPWV and ΔAIx were 0.4 m/sec and 3.3%, respectively

Heart rate, central systolic and diastolic BP, central pulse pressure, forward pressure, augmentation pressure, and AIx increased progressively from 1 min after handgrip exercise, and reached the maximal level at 2 min. Peripheral systolic BP, diastolic BP, pulse pressure and PWV also increased after 3-min handgrip exercise (Table 1). ΔPWV was significantly correlated with ΔAIx (*r* = 0.344, *P* = 0.037) (Fig. 2). The median values of ΔPWV and ΔAIx were 0.4 m/sec and 3.3%, respectively.

**Table 1:** Changes of hemodynamic parameters after isometric handgrip exercise (*N* = 37)

	Baseline	1 min	2 min	3 min
Heart rate (/min)	64.2 ± 9.2	67.5 ± 10.1*	68.9 ± 10.2*†	70.6 ± 12.9*†
Central systolic BP (mmHg)	118.4 ± 20.6	135.1 ± 21.4*	138.9 ± 22.5*†	140.4 ± 22.9*†
Central diastolic BP (mmHg)	63.4 ± 10.3	71.0 ± 10.6*	72.1 ± 11.3*†	72.7 ± 11.2*†
Central PP (mmHg)	55.0 ± 17.6	64.2 ± 18.2*	66.8 ± 19.2*†	67.7 ± 18.7*†
Forward pressure (mmHg)	108.7 ± 19.4	121.4 ± 20.7*	124.7 ± 21.8*†	126.2 ± 22.6*†
Augmentation pressure (mmHg)	9.7 ± 6.6	13.7 ± 8.3*	14.2 ± 8.5*	14.2 ± 8.7*
Augmentation index (%)	17.4 ± 10.5	21.2 ± 11.5*	21.5 ± 11.5*	21.4 ± 11.7*
Peripheral systolic BP (mmHg)	125.0 ± 20.4			140.7 ± 25.0*
Peripheral diastolic BP (mmHg)	62.2 ± 10.1			65.1 ± 10.3*
Peripheral PP (mmHg)	62.8 ± 18.4			75.7 ± 21.4*
Pulse wave velocity (m/sec)	9.99 ± 1.84			10.81 ± 2.33*

Values are mean ± standard deviation

*p < 0.05 vs. baseline, †*p* < 0.05 vs. 1 min

*BP* Blood pressure, *PP* Pulse pressure

Patients were divided into higher and lower ΔPWV (≥0.4 vs. < 0.4 m/sec) groups. Age, gender, BMI, smoker, co-morbid condition such as hypertension, DM, CKD or prior myocardial infarction, and medications were not significantly different between higher and lower ΔPWV groups except hyperlipidemia. Patients with higher PWV had more 2- or 3-vessel CAD (69% vs. 27%, *P* = 0.034). PCI was more frequently performed in patients with higher PWV (84% vs. 50%, *P* = 0.038) (Table 2). Baseline hemodynamic parameters including PWV and AIx were not significantly different between two groups. After 3-min handgrip exercise, PWV, Δcentral systolic BP, ΔAIx, and Δperipheral systolic BP were greater in patients with higher ΔPWV (Table 3).

**Table 2:** Comparison of baseline characteristics according to the changes of pulse wave velocity after isometric handgrip exercise

	All patients (*N* = 37)	ΔPWV ≥0.4 m/sec (*N* = 19)	ΔPWV < 0.4 m/sec (*N* = 18)	*P* value
Age (years)	63.3 ± 9.4	63.7 ± 8.4	62.9 ± 10.5	0.800
Male	23 (62)	14 (74)	9 (50)	0.184
Height (cm)	160.8 ± 8.9	162.0 ± 8.3	160.0 ± 9.6	0.422
Weight (kg)	65.7 ± 12.4	66.5 ± 12.1	64.9 ± 13.0	0.703
Body mass index (kg/m^2^)	25.27 ± 3.30	25.2 ± 3.0	25.3 ± 3.7	0.902
Smoker	9 (24)	5 (26)	4 (22)	0.459
Hypertension	26 (70)	14 (74)	12 (67)	0.728
Diabetes mellitus	8 (22)	5 (26)	3 (17)	0.693
Hyperlipidemia	33 (89)	19 (100)	14 (78)	0.046
Chronic kidney disease	19 (51)	9 (47)	10 (56)	0.746
Prior myocardial infarction	11 (30)	7 (37)	4 (22)	0.476
Coronary artery disease	27 (73)	16 (84)	11 (61)	0.151
CAG findings				0.051
1 vessel disease	13 (48)	5 (31)	8 (73)	0.034*
2 vessel disease	9 (33)	6 (38)	3 (27)	
3 vessel disease	5 (19)	5 (31)	0 (0)	
PCI	25 (68)	16 (84)	9 (50)	0.038
Medications				
Calcium channel blockers	16 (43)	10 (53)	6 (33)	0.325
RAS inhibitors	18 (49)	9 (47)	9 (50)	1.000
Beta-blockers	22 (60)	12 (63)	10 (56)	0.743
Nitrate	8 (22)	4 (21)	4 (22)	1.000
Statins	31 (84)	18 (95)	13 (72)	0.090

Values are mean ± standard deviation or number (%)

*1 vessel vs. 2- or 3-vessel disease

*CAG* Coronary angiography, *PCI* Percutaneous coronary intervention, *PWV* Pulse wave velocity, *RAS* Renin-angiotensin system

**Table 3:** Comparison of hemodynamic parameters according to the changes of pulse wave velocity after isometric handgrip exercise

	ΔPWV ≥0.4 m/sec (*N* = 19)	ΔPWV < 0.4 m/sec (*N* = 18)	*P* value
Isometric handgrip exercise			
Handgrip power, maximal (kg)	36.2 ± 13.2	31.0 ± 11.1	0.207
Handgrip power at exercise (kg)	13.3 ± 2.3	11.9 ± 3.3	0.134
Handgrip power %	38.9 ± 6.6	39.6 ± 5.8	0.736
Hemodynamic parameters, baseline			
Heart rate (/min)	62.5 ± 9.0	66.1 ± 9.3	0.240
Central systolic BP (mmHg)	114.3 ± 18.3	122.7 ± 22.4	0.216
Central diastolic BP (mmHg)	60.6 ± 7.1	66.4 ± 12.2	0.082
Central pulse pressure (mmHg)	53.7 ± 15.6	56.3 ± 19.8	0.660
Forward pressure (mmHg)	104.6 ± 17.3	113.1 ± 21.1	0.186
Augmentation pressure (mmHg)	9.7 ± 7.3	9.6 ± 5.9	0.974
Augmentation index (%)	17.9 ± 11.9	16.8 ± 9.2	0.742
Peripheral systolic BP (mmHg)	122.3 ± 19.4	127.8 ± 21.5	0.414
Peripheral diastolic BP (mmHg)	60.3 ± 7.7	64.2 ± 12.0	0.244
Peripheral pulse pressure (mmHg)	62.0 ± 17.3	63.6 ± 19.9	0.787
PWV (m/sec)	10.15 ± 2.07	9.83 ± 1.61	0.610
Exercise 3 min			
Heart rate (/min)	68.7 ± 13.9	72.7 ± 11.9	0.355
Central systolic BP (mmHg)	140.7 ± 20.9	140.1 ± 25.4	0.935
Central diastolic BP (mmHg)	70.7 ± 7.1	74.7 ± 14.2	0.289
Central pulse pressure (mmHg)	70.0 ± 18.0	65.3 ± 19.5	0.455
Forward pressure (mmHg)	124.1 ± 20.2	128.4 ± 25.3	0.567
Augmentation pressure (mmHg)	16.6 ± 9.3	11.7 ± 7.3	0.081
Augmentation index (%)	24.3 ± 12.4	18.3 ± 10.3	0.121
Peripheral systolic BP (mmHg)	144.3 ± 23.6	136.9 ± 26.5	0.377
Peripheral diastolic BP (mmHg)	65.7 ± 7.7	64.4 ± 12.7	0.708
Peripheral pulse pressure (mmHg)	78.6 ± 21.3	72.6 ± 21.6	0.395
PWV (m/sec)	12.03 ± 2.37	9.51 ± 1.44	< 0.001
Δ Central systolic BP (mmHg)	26.4 ± 10.3	17.3 ± 15.6	0.043
Δ Forward pressure (mmHg)	19.5 ± 7.5	15.3 ± 13.4	0.245
Δ Augmentation index (%)	6.3 ± 6.3	1.5 ± 6.8	0.033
Δ Peripheral systolic BP (mmHg)	22.1 ± 11.8	9.1 ± 8.7	0.001
Δ PWV (m/sec)	1.89 ± 1.00	−0.32 ± 0.49	< 0.001

Values are mean ± standard deviation

*BP* Blood pressure, *PWV* Pulse wave velocity

Patients were also divided into higher and lower ΔAIx (≥3.3 vs. < 3.3%) groups. Age, BMI, smoker, co-morbid condition such as hypertension, DM, hyperlipidemia, CKD, prior myocardial infarction, or CAD, and medications except gender were not significantly different between higher and low ΔAIx groups (Table 4). Patients with 2- or 3-vessel CAD were not significantly different (60% vs. 42%, *P* = 0.313), and PCI was similarly performed in both groups (68% vs. 67%, *P* = 1.000). Baseline central systolic BP and pulse pressure, augmentation pressure, AIx, peripheral systolic BP, and PWV were lower in higher ΔAIx group. However, after 3-min handgrip exercise, these hemodynamic parameters became not significantly different between both groups (Table 5).

**Table 4:** Comparison of baseline characteristics according to the changes of augmentation index after isometric handgrip exercise

	All patients (*N* = 37)	ΔAIx ≥3.3% (*N* = 19)	ΔAIx < 3.3% (*N* = 18)	*P* value
Age (years)	63.3 ± 9.4	61.7 ± 10.4	65.0 ± 8.1	0.288
Male	23 (62)	15 (79)	8 (44)	0.045
Height (cm)	160.8 ± 8.9	162.6 ± 8.4	158.9 ± 9.3	0.213
Weight (kg)	65.7 ± 12.4	69.2 ± 13.1	62.1 ± 10.8	0.081
Body mass index (kg/m^2^)	25.27 ± 3.30	25.98 ± 3.17	24.51 ± 3.36	0.180
Smoker	9 (24)	5 (26)	4 (22)	0.124
Hypertension	26 (70)	13 (68)	13 (72)	1.000
Diabetes mellitus	8 (22)	3 (16)	5 (28)	0.447
Hyperlipidemia	33 (89)	18 (95)	15 (83)	0.340
Chronic kidney disease	19 (51)	8 (42)	11 (61)	0.330
Prior myocardial infarction	11 (30)	5 (26)	6 (33)	0.728
Coronary artery disease	27 (73)	15 (79)	12 (67)	0.476
CAG findings				0.620
1 vessel disease	13 (48)	6 (40)	7 (58)	0.313*
2 vessel disease	9 (33)	6 (40)	3 (25)	
3 vessel disease	5 (19)	3 (20)	2 (17)	
PCI	25 (68)	13 (68)	12 (67)	1.000
Medications				
Calcium channel blockers	16 (43)	10 (53)	6 (33)	0.325
RAS inhibitors	18 (49)	9 (47)	9 (50)	1.000
Beta-blockers	22 (60)	13 (68)	9 (50)	0.325
Nitrate	8 (22)	4 (21)	4 (22)	1.000
Statins	31 (84)	18 (95)	13 (72)	0.090

Values are mean ± standard deviation or number (%)

*1 vessel vs. 2- or 3-vessel disease

*AIx* Augmentation index, *CAG* Coronary angiography, *PCI* Percutaneous coronary intervention, *RAS* Renin-angiotensin system

**Table 5:** Comparison of hemodynamic parameters according to the changes of augmentation index after isometric handgrip exercise

	ΔAIx ≥3.3% (*N* = 19)	ΔAIx < 3.3% (*N* = 18)	*P* value
Isometric handgrip exercise			
Handgrip power, maximal (kg)	37.0 ± 14.4	30.1 ± 8.7	0.089
Handgrip power at exercise (kg)	13.5 ± 2.8	11.7 ± 2.7	0.060
Handgrip power %	38.7 ± 6.7	39.8 ± 5.6	0.566
Hemodynamic parameters, baseline			
Heart rate (/min)	65.7 ± 9.9	62.7 ± 8.4	0.334
Central systolic BP (mmHg)	109.9 ± 16.2	127.3 ± 21.3	0.008
Central diastolic BP (mmHg)	62.0 ± 8.4	65.0 ± 12.0	0.373
Central pulse pressure (mmHg)	48.0 ± 14.7	62.3 ± 17.7	0.011
Forward pressure (mmHg)	103.7 ± 15.8	114.1 ± 21.9	0.105
Augmentation pressure (mmHg)	6.2 ± 4.9	13.3 ± 6.2	< 0.001
Augmentation index (%)	13.3 ± 9.6	21.7 ± 10.0	0.013
Peripheral systolic BP (mmHg)	118.5 ± 19.3	131.8 ± 19.8	0.045
Peripheral diastolic BP (mmHg)	61.3 ± 8.3	63.2 ± 11.9	0.562
Peripheral pulse pressure (mmHg)	57.2 ± 17.8	68.6 ± 17.6	0.058
Pulse wave velocity (m/sec)	9.30 ± 1.72	10.72 ± 1.72	0.017
Exercise 3 min			
Heart rate (/min)	70.5 ± 13.4	70.8 ± 12.9	0.948
Central systolic BP (mmHg)	135.0 ± 19.7	146.1 ± 25.1	0.140
Central diastolic BP (mmHg)	71.5 ± 7.2	73.9 ± 14.4	0.527
Central pulse pressure (mmHg)	63.5 ± 18.7	72.2 ± 18.0	0.157
Forward pressure (mmHg)	120.9 ± 17.0	131.7 ± 26.7	0.154
Augmentation pressure (mmHg)	14.1 ± 9.1	14.4 ± 8.5	0.908
Augmentation index (%)	22.2 ± 11.6	20.4 ± 12.0	0.648
Peripheral systolic BP (mmHg)	136.2 ± 22.6	145.6 ± 27.1	0.259
Peripheral diastolic BP (mmHg)	64.5 ± 7.6	65.6 ± 12.8	0.754
Peripheral pulse pressure (mmHg)	71.6 ± 21.1	79.9 ± 21.4	0.242
Pulse wave velocity (m/sec)	10.68 ± 2.59	10.94 ± 2.10	0.747
Δ Central systolic BP (mmHg)	25.1 ± 11.7	18.8 ± 15.3	0.169
Δ Forward pressure (mmHg)	17.2 ± 7.8	17.7 ± 13.6	0.900
Δ Augmentation index (%)	9.0 ± 5.3	−1.3 ± 3.8	< 0.001
Δ Peripheral systolic BP (mmHg)	17.7 ± 11.2	13.7 ± 13.1	0.330
Δ Pulse wave velocity (m/sec)	1.38 ± 1.39	0.21 ± 1.08	0.008

Values are mean ± standard

*AIx* Augmentation index, *BP* Blood pressure

**Fig. 3:**
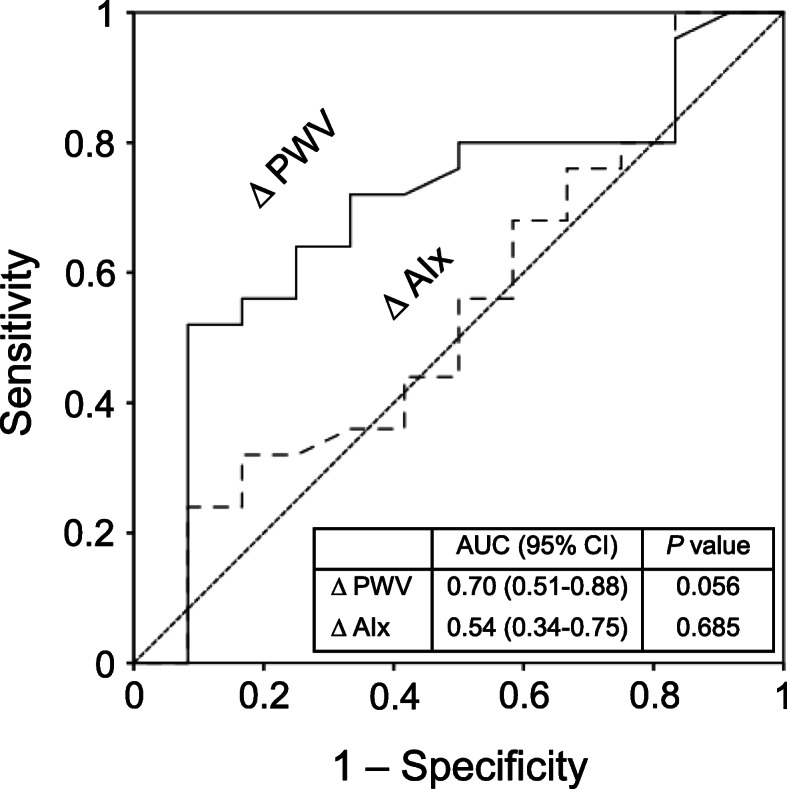
Area under curve (AUC) with 95% confidence interval (CI) of Δpulse wave velocity (PWV) or Δaugmentation index (AIx) in association with percutaneous coronary intervention by C-statistics

C-statistics showed the area under curve (AUC) of ΔPWV in association with PCI was 0.70 (95% confidence interval [CI] 0.51–0.88; *P* = 0.056). In contrast, it was 0.54 (95% CI 0.34–0.75; *P* = 0.685) in case of ΔAIx (Fig. 3). In multiple logistic regression analysis after adjustment, ΔPWV was significantly associated with PCI (odds ratio [OR] 7.78; 95% CI 1.26–48.02; *P* = 0.027), but ΔAIx was not (OR 2.81; 95% CI 0.37–21.30; *P* = 0.318).

## Discussion

The main result of this study is that, although a significant correlation was observed between ΔPWV and ΔAIx after isometric handgrip exercise, only higher ΔPWV was associated with the lesion extent of CAD and the necessity for coronary revascularization.

Atherosclerosis is a generalized process of the arterial system including not only coronary arteries but also larger arteries. The atherosclerotic change of the aorta makes the arterial system stiffer, and the stiff aorta is associated with myocardial ischemia and incident CAD [7–9]. Therefore, indexes of arterial stiffness are usually increased in patients with CAD. However, these indexes are often increased in elderly without CAD because systolic BP increases with aging, there is a close association between systolic BP and PWV, and both vascular aging and hypertension are the powerful factors determining arterial stiffness [1]. As a result, the difference of indexes of arterial stiffness between patients with and without CAD may be not evident in elderly. The previous meta-analysis showed that aortic PWV was a predictor of future cardiovascular events such as CAD or stroke even in elderly, but its relation to cardiovascular events was weaker in older than younger patients, and the hazard ratio was decreased with age (1.89, 1.77, 1.36, and 1.23 for age ≤ 50, 51–60, 61–70, and > 70 years, respectively) [9]. The usefulness of AIx as a marker for CAD in older patients is even more unclear. Because AIx strongly correlates with age and it is also influenced by heart rate, body length or the pattern of ventricular ejection [3], AIx may not be an accurate index for arterial stiffness. The comparison of aortic PWV with AIx as a surrogate for the extent and severity of CAD was possible in one study that measured both indexes in the same patients at the time of coronary angiography. It showed the significant association of the extent and severity of CAD with PWV, but not with AIx [12].

Isometric handgrip exercise is a non-invasive and easily applicable maneuver to increase heart rate, BP and left ventricular afterload [21–23]. In this study, central systolic BP, forward and augmentation pressures, AIx, peripheral BP and PWV were increased after handgrip exercise. Although ΔPWV and ΔAIx correlated each other, the pattern of ΔPWV and ΔAIx after handgrip exercise was different. Baseline PWV was not different between higher and lower ΔPWV groups, but after 3-min handgrip exercise, Δcentral systolic BP, Δperipheral systolic BP and PWV were greater in higher ΔPWV group. However, baseline AIx was smaller in higher ΔAIx group, and AIx after 3-min handgrip exercise became similar between higher and lower ΔAIx groups because of more increase of augmentation pressure in lower ΔAIx group. All of these findings suggest that ΔAIx may be larger in patients with lower baseline AIx regardless of arterial stiffness and reach plateau after handgrip exercise. Therefore, ΔAIx after handgrip exercise may not be as a good index for arterial stiffness as ΔPWV.

The mean age of patients of this study was 63.3 years, 65% were > 60 years and 70% of them had hypertension. These findings may be usually seen in patients with CAD. In this clinical setting, simple PWV value may not differentiate patients with CAD and the lesion severity of CAD. In the previous study, we showed that PWV value at rest was not different between patients with and without CAD, but after 3-min handgrip exercise, PWV was significantly increased only in patients with CAD [24]. However, the change of PWV after handgrip exercise showed a diverse pattern such that PWV increased even in patients without CAD, and a cut-off point of ΔPWV after handgrip exercise, which was associated with the lesion extent of CAD and the necessity for coronary revascularization, was not evaluated in the previous study. In this study, higher ΔPWV group had more 2- or 3-vessel CAD and underwent more PCI than lower ΔPWV group. On the other hand, the lesion extent of CAD and the necessity for PCI were not different between higher and lower ΔAIx groups. In addition, C-statistics and multiple logistic regression analysis showed that ΔPWV was associated with PCI but not ΔAIx. These findings suggest that ΔPWV may be used as an index for differentiating the lesion extent of CAD and the necessity for coronary revascularization even in elderly patients with hypertension, but ΔAIx may not.

This study has several limitations. First, a fluid-filled catheter system, instead of a high-fidelity micromanometer, was used to record the pressure waveforms. A definite merging point of the forward and the reflected waves on the recorded pressure waveform was not identified in aortic pressure waveforms of 4 patients (9.5%). Second, over half of patients were taking medications with a vasodilation property, which may influence the change of BP, PWV, and AIx after handgrip exercise. However such medications were equally taken in both higher and lower ΔPWV or ΔAIx groups, and baseline central and peripheral BP were not different between groups. Third, PCI performed not only on the day of the study but also 1 year ago was counted as having coronary revascularization. Including only prospectively conducted coronary revascularization would have increased the feasibility of the study. Fourth, the number of patients might not be enough to accurately evaluate the changes of PWV and AIx after handgrip exercise. Nevertheless, the association between higher ΔPWV and the lesion extent of CAD or the necessity for PCI was demonstrated.

This was an invasive study that measured hemodynamic parameters during CAG. To be widely use, the applicability of a non-invasive method measuring ΔPWV or ΔAIx after handgrip exercise using an applanation tonometry needs to be validated in a future study.

## Conclusions

Both PWV and AIx increased after isometric handgrip exercise, and ΔPWV was significantly correlated with ΔAIx. However, higher ΔPWV was associated with the lesion extent of coronary atherosclerosis and the necessity for coronary revascularization, but higher ΔAIx was not.

## Data Availability

The datasets used and/or analyzed during the current study are available from the corresponding author on reasonable request.

## Abbreviations

AIx: Augmentation index
AP: Augmentation pressure
CAD: Coronary artery disease
CAG: Coronary angiography
PCI: Percutaneous coronary intervention
PWV: Pulse wave velocity
RAS: Renin-angiotensin system
